# mTORC1/2 inhibition re-sensitizes platinum-resistant ovarian cancer by disrupting selective translation of DNA damage and survival mRNAs

**DOI:** 10.18632/oncotarget.25869

**Published:** 2018-09-04

**Authors:** Gizelka David-West, Amanda Ernlund, Abhilash Gadi, Robert J. Schneider

**Affiliations:** ^1^ Division of Gynecologic Oncology, New York University School of Medicine, New York, NY, USA; ^2^ Department of Microbiology, New York University School of Medicine, New York, NY, USA; ^3^ Perlmutter Cancer Center, New York University School of Medicine, New York, NY, USA; ^4^ New York Medical College, Westchester Medical Center, Hawthorne, NY, USA

**Keywords:** mTOR, ovarian cancer, platinum resistance, translational regulation

## Abstract

Platinum resistance is a major cause of treatment failure and mortality in epithelial ovarian cancer. mTORC1/2 inhibitors, which impair mRNA translation, can re-sensitize resistant ovarian cancer cells to platinum chemotherapy but the mechanism remains poorly described. Using platinum-resistant OVCAR-3 cells treated with the selective mTORC1/2 inhibitor INK128/MLN128, we conducted genome-wide transcription and translation studies and analyzed the effect on cell proliferation, AKT-mTOR signaling and cell survival, to determine whether carboplatin resistance involves selective mRNA translational reprogramming, and whether it is sensitive to mTORC1/2 inhibition. Gene ontology and Ingenuity Pathway Analysis (IPA) were used to categorize gene expression changes into experimentally authenticated biochemical and molecular networks. We show that carboplatin resistance involves increased mTORC1/2 signaling, resulting in selective translation of mRNAs involved in DNA damage and repair responses (DDR), cell cycle and anti-apoptosis (survival) pathways. Re-sensitization of ovarian cancer cell killing by carboplatin required only modest mTORC1/2 inhibition, with downregulation of protein synthesis by only 20-30%. Genome-wide transcriptomic and translatomic analyses in OVCAR-3 cells revealed that the modest downregulation of global protein synthesis by dual mTORC1/2 inhibition is associated with greater selective inhibition of DDR, cell cycle and survival mRNA translation, which was confirmed in platinum-resistant SKOV-3 cells. These data suggest a clinical path to re-sensitize platinum resistant ovarian cancer to platinum chemotherapy through partial inhibition of mTORC1/2, resulting in selective translation inhibition of DDR and anti-apoptosis protective mRNAs.

## INTRODUCTION

Ovarian cancer is the second most common gynecologic malignancy, and the number one cause of death among all gynecologic malignancies [1]. The high death rate is attributed to the fact that approximately 75% of cases are diagnosed at an advanced stage, contributing to the poor overall 5 year survival rate of 45% [2]. Platinum-based chemotherapy is the standard of care for ovarian cancer. While excellent in response initially, most patients ultimately relapse with platinum-resistant disease, and approximately 25% of patients acquire de novo resistance during primary treatment or relapse within 6 months [reviewed in 3]. In the recurrent setting, there are no truly effective options for women with platinum resistant disease. Recent research and analysis of ovarian cancer genomic alterations derived from the Cancer Genome Atlas (TCGA) project call attention to the need for new molecular targets by which treatment responses can be improved in the recurrent, platinum-resistant setting [4-6].

Standard therapy for ovarian cancer primarily comprises platinum based chemotherapeutic agents administered in combination with a taxane, a tubulin stabilizing drug [7]. Neoadjuvant chemotherapy may also be given, although median disease-free survival is still only 12 to 24 months [8-10] and the majority of patients will recur, making the management of recurrent disease a major challenge. In the platinum-resistant recurrent setting, no therapies are curative [11].

Genomic analyses suggest that different histologic subtypes of ovarian cancer have different genetic alterations and deregulated signaling pathways that might be therapeutically targeted [4, 12]. Specifically, the PI3K/AKT/mTOR pathway is commonly upregulated with increased activation in most subtypes of epithelial ovarian carcinoma, including approximately 50% of high-grade serous ovarian carcinomas [4, 13]. Genetic alterations are found in various components of this pathway such as PI3KCA (PI3K) gain of function mutations, AKT2 amplifications, loss of PTEN expression (a negative phosphatase regulator of PI3K activity), and activating mutations that signal to AKT and mTOR [4, 14, 15]. All of these alterations are integral components of mTOR pathway activation, which can lead to increased protein synthesis, and specifically increased translation of certain mRNAs, such as those encoding angiogenic and pro-proliferative functions [15, 16]. Major oncogenic signaling pathways converge on protein synthesis at the level of the protein kinase mTOR, a key regulator of cellular metabolism, autophagy, mRNA translation and cell motility.

With a significant role in tumorigenesis, including that of ovarian cancer, inhibition of the mTOR pathway has been extensively investigated in the preclinical and clinical settings. mTOR forms two protein complexes with different activities, mTOR complex 1 (mTORC1) and mTOR complex 2 (mTORC2). mTORC1 consists of mTOR, Raptor and GβL proteins among others [17], and is a major regulator of cap-dependent mRNA translation through its phosphorylation (inactivation) of the 4E-BP family, negative regulators of translation initiation factor eIF4E [18]. mTORC2 consists of mTOR, Rictor and GβL proteins among others. mTORC2 phosphorylates and activates pro-oncogenic AKT at serine 473, leading to increased cancer cell proliferation and survival [19]. Moreover, pro-oncogenic AKT activity is increased by positive feedback through S6K/IRS1 from loss of mTORC1 during inhibition with rapalogs (everolimus, temsirolimus, sirolimus) [20-22].

Rapalog inhibitors of mTORC1 are allosteric inhibitors of the FKBP12 mTOR chaperone and therefore act upstream of mTORC1. They have shown limited activity in clinical trials, poor response and rapid development of tumor resistance, in part because they only poorly inhibit mTORC1, and because they release feedback upregulation of AKT [20, 21, 23, 24]. Consequently, direct acting ATP-active site inhibitors of mTOR have been developed that effectively block both mTORC1 and mTORC2 activities [17, 25, 26]. Dual mTORC1/2 inhibition overcomes feedback activation of PI3K and AKT pathways, potentially resulting in more effective anti-tumor activity. Because mTORC1/2 is at the crossroads of a number of oncogenic signaling pathways, including the MAPK/ERK and PI3K/AKT pathways, as well as many receptor tyrosine kinases, and is therefore strongly activated by multiple oncogenic signals, there is strong interest in its inhibition in conjunction with existing chemotherapeutics.

In *in vitro* and *in vivo* mouse models of platinum resistant high grade papillary serous ovarian cancer, we and others have demonstrated notable tumor growth inhibition in models when blocking mTORC1/2 compared to mTORC1 alone, plus greater anti-proliferative effects when combined with carboplatin, and decreased phosphorylation of select DNA repair proteins [27-29]. These results demonstrated reversal of platinum resistance with mTORC1/2 inhibition but did not address the molecular mechanism involved. Clinical experience with mTOR inhibitors in ovarian cancer have to date been derived from the use of rapalogs that inhibit only mTORC1 in early stage clinical studies. We therefore sought to investigate the mechanism for platinum re-sensitization by mTORC1/2 inhibition using the clinically available inhibitor INK128/MLN128, in platinum-resistant ovarian cancer cells. We show that platinum resistance of ovarian cancer cells can be reversed by inhibition of mTORC1/2 and involves the greater translational inhibition of specific mRNAs encoding survival, cell cycle and DDR functions. These findings suggest the synergistic use of mTORC1/2 inhibitors with genotoxic DNA damage agents should be explored in the clinical setting in platinum-resistant ovarian cancer.

## RESULTS

### mTORC1/2 inhibition blocks proliferation and promotes platinum re-sensitization

**Figure 1:**
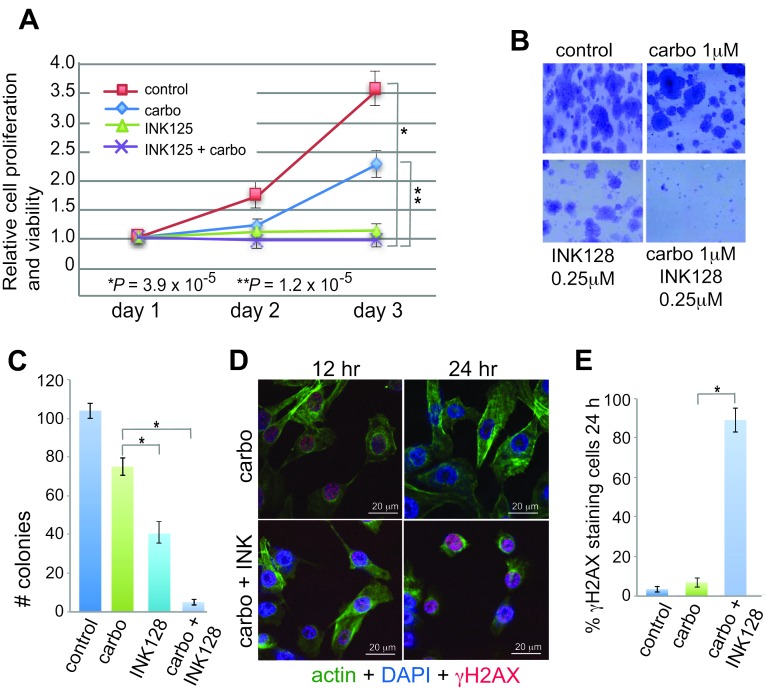
Anti-proliferative and sensitizing effects of INK128 with carboplatin in OVCAR-3 cells **A.** Proliferation/survival assay was performed in triplicate and mean dye absorbance values from MTT for each treatment were recorded and normalized to the mean dye absorbance value of media only wells. Statistical analysis by ANOVA. **B.** Representative images of clonogenic cell survival assays using 600 cells/well in 6 well plates. **C.** Quantification of clonogenic cell survival assays performed in triplicate, repeated 5 times at drug doses as shown in (B). **D.** DNA-damage response signaling in 1 µM carboplatin treated and 1 µM carboplatin/0.25 µM INK128 treated cells. Cells were pre-treated with INK128 for 4 h, INK128 maintained and carboplatin added for 5 h, and cells subjected to direct immunofluorescence analysis at 12 h and 24 h. Cells stained with DAPI (blue), FITC-actin (green), γH2AX (H2AX-S139P, red). γH2AX staining consisted of primary monoclonal antibody and TRITC anti-mouse secondary antibody. Representative images shown; scale bar 20 µm. **E.** Quantification of results shown in (D) representative images obtained from 5 fields chosen at random with ≥50 cells/field. *, *P* < 0.01 by paired Student *t*-test.

Studies were conducted using GI_50_ concentrations of INK128 and a low dose of carboplatin (1 µM) that does not affect OVCAR-3 cell proliferation or survival [13, 30, 31]. Cell proliferation/viability and clonogenic assays were used to measure anti-proliferative and platinum-sensitizing effects of INK128 (Figure 1). While dose titrations are not shown, we found the lowest effective dose of INK128 to be 0.25 µM and of carboplatin to be 1.0 µM, defined as that which effectively inhibited clonogenic cell survival, consistent with published studies [13, 30, 31]. We therefore carried out all studies in OVCAR-3 cells at these dose levels. Although mTORC1/2 inhibition alone blocks cell proliferation (Figure 1A), clonogenic cell survival analysis shows a qualitative reduction in colony size (Figure 1B) and a statistically significant reduction in colony number due to inhibition of proliferation by INK128 (Figure 1C), but only when treated with the combination of carboplatin and INK128 was there a severe reduction (>95%). These data suggest that the combination treatment increased inhibition of cell growth (size), and viability, whereas cell proliferation was already fully blocked by mTORC1/2 inhibition alone with INK128. Independent confirmation of these data was obtained by staining cells for γH2AX, which decorates double-strand DNA (dsDNA) breaks (Figure 1D, 1E). Carboplatin alone induced light staining at 12 h that was cleared by 24 h, indicative of strong resistance to drug-mediated genotoxic DNA damage, and effective DNA repair, as expected. In contrast, co-treatment with carboplatin and INK128 resulted in significant dsDNA breaks at 12 h, shown by γH2AX staining, which persisted and increased by 24 h, consistent with re-sensitization to DNA damage and an impaired ability to repair dsDNA lesions.

**Figure 2:**
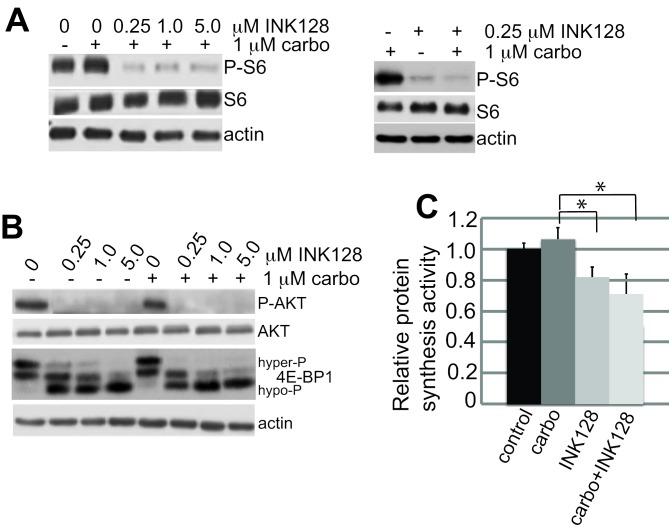
mTORC1/2 inhibition by INK128 is unaffected by carboplatin treatment **A.**, **B.** Samples consisting of equal protein amounts from total cell lysates were resolved by SDS-PAGE and detected by immunoblot. Cells were treated with carboplatin and INK128 as described in the legend to Figure 1. Immunoblots are shown at 24 h post-treatment. Representative blots of 3 independent studies. **C.** Cells were labeled during indicated drug treatments with 25 µCi of [^35^S]-methionine-cysteine/mL and specific activity of labeled protein incorporation into nascent protein determined by TCA precipitation. The mean of 3 studies was normalized to controls, with standard error of the mean (SEM) shown. Statistical analysis by paired Student *t*-test. *, *P* < 0.01.

Immunoblot analysis demonstrated that treatment with mTORC1/2 inhibitor INK128 alone at 0.25 µM fully blocked ribosomal S6 (rS6) protein phosphorylation (Figure 2A) but only partially blocked 4E-BP1 phosphorylation, shown by the partial shift to more hypo-phosphorylated forms (Figure 2B). The greater sensitivity of ribosomal S6 protein phosphorylation to mTOR inhibition compared to 4E-BP1 is well established [17, 19, 24]. There was no additional effect in combination with carboplatin (Figure 2A, 2B). The overall effect on protein synthesis was assessed by metabolic protein synthesis specific activity, determined by ^35^S-methionine incorporation (Figure 2C). There was a 20-30% reduction in overall protein synthesis at the lowest effective dose of INK128 used in these studies (0.25 µM), which was not statistically altered by addition of carboplatin (although there was a trend toward a slight further reduction of protein synthesis), consistent with the partial inhibition of 4E-BP1 phosphorylation. The lack of a strong effect of carboplatin treatment on protein synthesis and mTOR signaling is consistent with its specific induction of DNA damage. The synergistic reduction in carboplatin resistance and ovarian cancer cell viability with combined carboplatin and INK128 treatment was only observed in extended colony formation assays because they combine the cumulative effect of DNA damage over days with partially impaired protein synthesis, which is not captured in short-term cell proliferation/viability studies. These data also indicate that the translation reduction by combined treatment is due entirely to mTORC1/2 inhibition by INK128. We therefore asked whether re-sensitization of platinum-resistant OVCAR-3 cells results from the 20-30% reduction in global protein synthesis or involves selective translational inhibition of specific mRNAs.

### Genome-wide transcription and translation analysis identifies selectively translated mRNAs in platinum resistance

**Figure 3:**
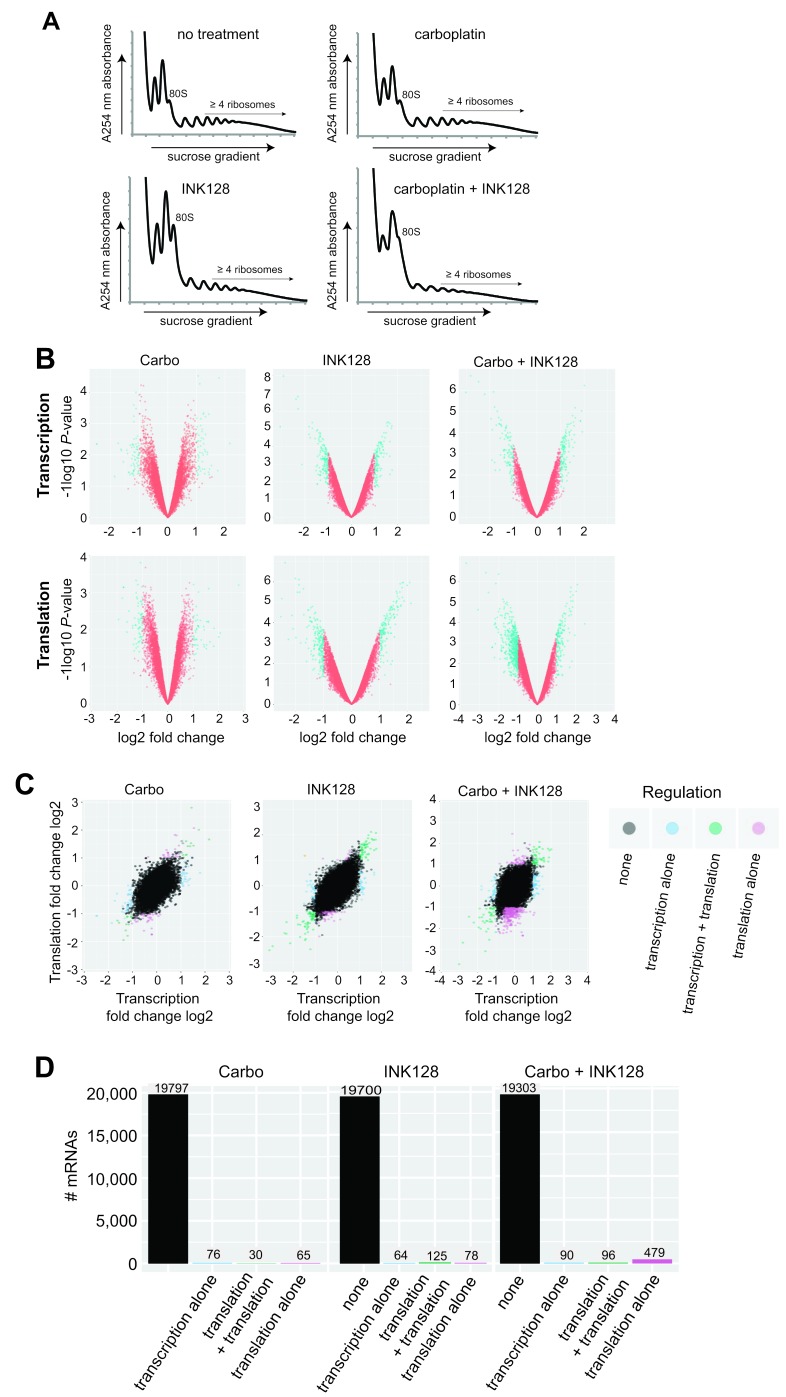
Transcriptomic and translatomic analysis of carboplatin re-sensitization by mTORC1/2 inhibition **A.** Absorbance profiles of ribosome subunits and polysomes for untreated and all treatment groups. OVCAR-3 cells were treated with DMSO as control, 1 µM carboplatin, 0.25 µM INK128, or both, as described in Material and Methods. Cycloheximide (100 µg/ml) was added, polysomes prepared and sorted by centrifugation through 10-50% sucrose gradients using equal RNA amounts and equal volume fractions collected while simultaneously monitoring absorbance at 254 nm, as described [38]. **B.** Volcano plots demonstrating transcriptional and translational alterations plotted as log_2_ fold changes against log_10_ *P*-values for all three treatment conditions. **C.** Log_2_ scatter plots of transcriptomic and translatomic results for all treatment groups (carboplatin alone, INK128 alone, combination treatment) analyzed for altered transcription, transcription + translation, or translation alone (translation efficiency). Two complete sets of independently performed studies were used to develop transcriptome and translatome data sets for analysis. **D.** Histogram representation of number of mRNAs out of total mRNAs altered in all three treatment conditions for transcription, transcription + translation, or translation alone (translation efficiency).

We investigated whether inhibition of mTORC1/2 and its ability to re-sensitize resistant ovarian cancer cells to platinum therapy is linked to changes in selective mRNA translation. To do so, we carried out simultaneous genome-wide transcriptomic and translatomic analysis of OVCAR-3 cells blocked in mTORC1/2 with 0.25 µM INK128 in the presence or absence of carboplatin, performed at 24 h, long before cells undergo programmed death (typically at ∼72 h). Total mRNA levels were compared to mRNA levels in the well-translated (≥4 ribosome) fraction, obtained by sorting polyribosomes by sucrose gradient density centrifugation for untreated, single agent treated and combination treated cells (Figure 3A; Supplementary data sets 1 and 2). Ribosome density is an established surrogate for translation activity. Polysomal profiling showed only a moderate reduction in mRNA-ribosome content with mTORC1/2 inhibition, which was slightly further reduced in carboplatin treated cells which may be reflective of their greater stress (Figure 3A), consistent with the profile in overall protein synthesis shown earlier by metabolic labeling. We analyzed three sets of conditions to fully explore the genome-wide changes in mRNA abundance and translation: (1) expression levels of total mRNA (primarily transcription activity); (2) mRNA polysome association, regardless of whether changes are due to mRNA abundance or translational regulation; and (3) ratio of heavy polysome mRNA/total mRNA, which measures stronger translation-specific changes (translation efficiency). Analyses for the two independent studies used thresholds for transcription and translation based on values determined from the distribution plots of each, which were 2-fold for total mRNA (log_2_ = 1.0), and 1.5-fold for heavy polysome association (log_2 =_ 0.6). The lower cutoff value for genome-wide translation is acceptable because small changes in protein expression can have large physiological effects. Significance was set at *P* < 0.05 for all analyses. Array analyses were performed with background correction and normalization, followed by filtering for low expression, and the ratio of translation/transcription plotted as independent variables. Limma, a platform for analysis of gene expression data from microarrays, was utilized for extraction of differential translation data.

Figure 3B volcano plots present genome-wide transcription and translation data comparing *P*-values across fold changes, whereas Figure 3C scatter plots present fold changes comparing transcription to translation, across all three conditions (carboplatin alone, INK128 alone, combination). Carboplatin alone produced few changes in transcription or translation, averaging just 76 mRNAs altered in abundance and 65 mRNAs altered in translation alone (Figure 3C, 3D). Inhibition of mTORC1/2 with INK128 at 20-30% reduction in overall protein synthesis also did not result in large numbers of mRNAs transcriptionally or translationally altered (Figure 3C, 3D). However, when mTORC1/2 inhibition by INK128 was combined with carboplatin there was a much greater reduction in the number of mRNAs undergoing translation (479), which likely reflects translational inhibition of mRNAs that are only moderately transcriptionally induced (<2 fold) by carboplatin-mediated DNA damage, and therefore not captured in the transcription analysis. Thus, the combination treatment of mTORC1/2 inhibition (INK128) with carboplatin generated the greatest number of mRNAs that were differentially translated (mostly downregulated), indicating that the combined treatment produces more specific mRNA translational regulation than INK128 alone or carboplatin alone.

### Molecular and biochemical pathway analysis of mRNAs selectively inhibited in translation by mTORC1/2 blockade

**Figure 4:**
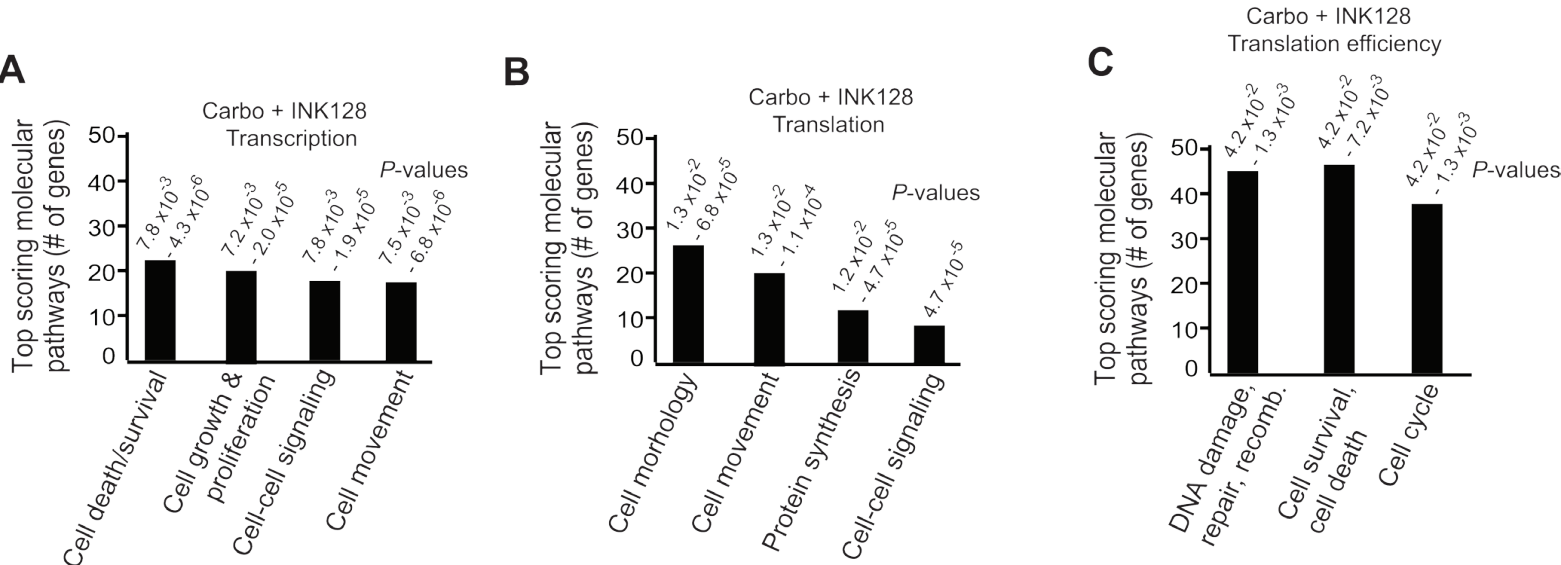
Top scoring molecular pathways for transcription, translation and translation efficiency by Ingenuity Pathway Analysis for carboplatin + INK128 treated OVCAR-3 cells Results represent the number of mRNAs scoring in each of the top ranked pathways and the range of *P*-values. **A.** Transcription analysis: mRNAs altered in abundance alone. **B.** Translation analysis: mRNAs altered in heavy polysome association (≥4 ribosome heavy polysome/total mRNA) regardless of total mRNA abundance. **C.** Translation efficiency analysis: ratio of heavy polysome/total mRNA.

Using Ingenuity Pathway Analysis (IPA) we categorized major gene expression changes for transcription alone, translation (transcription and polysomal mRNA abundance) and translation efficiency (translation-only, polysome mRNA abundance/total mRNA abundance, an authentic measure of selective mRNA translation) in combined carboplatin/INK128 treated cells. These data were categorized into experimentally authenticated biochemical-molecular pathways, classifying them into biologically significant functions (Figure 4A-4C). Data output was represented as a specific biological pathway/process based on the up- or down-representation of mRNAs on the list and *P*-value ranges. Transcriptional changes resulting from combined carboplatin/INK128 treatment were enriched for a fairly small number of cell death and survival, cell proliferation and cell-cell signaling mRNAs (Figure 4A). Translational changes, which are not corrected for transcription-driven translation changes, also identified a small number of altered mRNAs, interestingly, few of which were the basis for DNA damage/repair and cell survival categories (Figure 4B). In contrast, when categorized by translation efficiency, three major pathways were highly represented by combined carboplatin-INK128 treatment. Notably, there were a larger number of mRNAs that showed translation-specific down-regulation, in association with re-sensitization of OVCAR-3 cells when treated with INK128 and carboplatin (Figure 4C). Top scoring molecular-cellular pathways for translation efficiency were dominated by DNA damage and repair function mRNAs, anti-apoptosis and survival function mRNAs, and cell cycle function mRNAs. These data and categories suggest a model of synergistic activities that are specifically increased through selective mRNA translation by increased mTORC1/2 activity with carboplatin-resistance in ovarian cancer cells, which can be selectively impaired by partial mTORC1/2 inhibition and result in re-sensitization to genotoxic DNA damage by carboplatin.

Filtered lists of specific translationally reduced mRNAs derived from translation efficiency data with combined carboplatin-INK128 treatment were generated, ranked by log_2_ fold reduction. Consistent with IPA analysis, the largest selective reductions in mRNA translation by combined carboplatin and INK128 treatment were found to correspond to mRNAs involved in promoting DNA repair, survival and regulation of cell cycle (Tables 1, 2). For example, strong translational down-regulation of the mRNAs involved in DNA replication/repair, chromosome segregation and cell proliferation, a variety of cell survival functions (such as oxidative stress), and others were identified. Among these were several cIAP proteins (e.g., BIRC5, BIRC3/survivin), caspase inhibitors (e.g., CAAP1), promoters of oxidative stress resistance (e.g., OXR1, TXN), and a number of DNA break repair and DNA synthesis proteins, including polymerases and ribosomal proteins.

**Table 1:** DDR and cell cycle mRNAs translationally impaired by INK128 + carboplatin treatment

Gene name	Log2 fold reduction	Function
PTMA	-2.80	Thymosin A1: involved in chromatin remodeling, DNA replication, has anti-apoptotic function as well
UQCRFS1	-2.60	Ubiquitinol Cyt C reductase: iron sulfur polypeptide in mitochondrial respiratory chain, ATP synthesis
TAF1D	-2.41	RNA POL1 TATA-box binding factor, rRNA synthesis
RAD17	-2.22	Required for cell cycle arrest during DDR
OXR1	-1.94	Oxidative stress resistance, transcriptional stress network regulator
SUMO2	-1.81	Sumoylation protein, important in DDR pathway function
TXN	-1.52	Thioredoxin: redox signaling, reduce oxidative stress during stress responses, cell survival during stress
MIS12	-1.36	Kinetochore complex protein, promotes proper mitotic chromosome alignment
CEP70	-0.93	Regulates microtubule assembly, promotes resistance to microtubule assembly drugs
FGF9	-1.14	Stimulates cell proliferation, cell survival
CENPH	-1.12	Kinetochore and centromere protein, involved in sister chromatid separation during DNA replication
CETN3	-1.00	Centrin-3: chromosome duplication and separation
ESCO-1	-1.00	N-acetyl transferase: chromatid cohesion and DNA replication
RAD51C	-1.00	dsDNA break repair
CDKN2	-1.00	CDK4 inhibitor, prevents unauthorized cell cycle progression, block cell cycling during DNA damage
ESCO1	-1.00	Sister chromatid adhesion
CASC1	-1.00	Cancer susceptibility
CDK7	-0.95	Promotes G2-M transition
POLR1D	-0.95	Required for rRNA synthesis
POLQ	-0.92	Essential for DNA repair during DNA damage and restricted homologous recombination repair pathway
CIRBP1	-0.94	Cold inducible RNA binding protein, protects against DNA damage during stress
POLB	-0.93	DNA base-excision repair, DDR
FBXW7	-0.92	F-box ubiquitination protein. Cyclin E target. Protective against ovarian cancer.
RALB	-0.88	Multifunctional GTPase, involved in signaling, cell proliferation activation

**Table 2:** Survival and anti-apoptotic mRNAs translationally impaired by INK128 + carboplatin treatment

Gene name	Log2 fold reduction	Function
RPL21P28	-1.70	Ribosome large subunit protein
RPL21	-1.50	Ribosome large subunit protein, association with increased cMyc expression
BIRC5	-1.48	cIAP5, survivin, inhibitor of apoptosis, driver of DNA damage drug resistance
RPL27A	-1.47	Ribosome large subunit protein
RPL9	-1.37	Ribosome large subunit protein
BIRC3	-1.35	cIAP2 protein, inhibitor of apoptosis, driver of DNA damage drug resistance
RPS15A	-1.35	Ribosome small subunit protein
RPS21	-1.28	Ribosome small subunit protein
RPS19	-1.21	Ribosome small subunit protein, higher expression linked to certain carcinomas and Diamond-Blackfan anemia
PHF5A	-1.17	Involved in splicing factor 3b complex, transcriptional elongation, pluripotency maintenance
SPATA2	-1.17	Recruits and activates deubiquitinases, regulates inflammatory signaling, loss promotes necroptosis by TNF
RPL11	-1.10	Ribosome large subunit protein, blocks mdm2 degradation of p53, involved in cell cycle arrest during stress, DDR
RPL22L1	-1.03	Ribosome large subunit protein, regulates pre-mRNA splicing, ribosome biogenesis
CCN1/CYR61	-1.07	Promotes cancer cell survival, proliferation, angiogenesis
AVEN	-0.96	Inhibitor of caspases and apoptosis, blocks cell cycle during DDR, loss promotes genomic instability and cell death
BECN1	-0.90	Binds BCL2, promotes ovarian cancer and evades apoptosis with increased autophagy
RPL17	-0.90	Ribosome large subunit protein, involved in large subunit biogenesis
PNO1	-0.90	Involved in 18S rRNA processing, ribosomal and proteasome biogenesis
RPL23A	-0.88	Ribosome large subunit protein
PIGA	-0.87	Involved in GPI anchor adhesion, GPI function in signaling
RPS3A	-0.83	Ribosome small subunit protein, overexpression associated with carcinoma
METAP2	-0.83	Promotes N-terminal methionine removal, angiogenesis, prevents eIF2α phosphorylation and inhibition of protein synthesis during cell stress
RPL37	-0.83	Ribosome large subunit protein, overexpressed in certain cancers
CAAP1	-0.80	Caspase and apoptosis inhibitor

**Figure 5:**
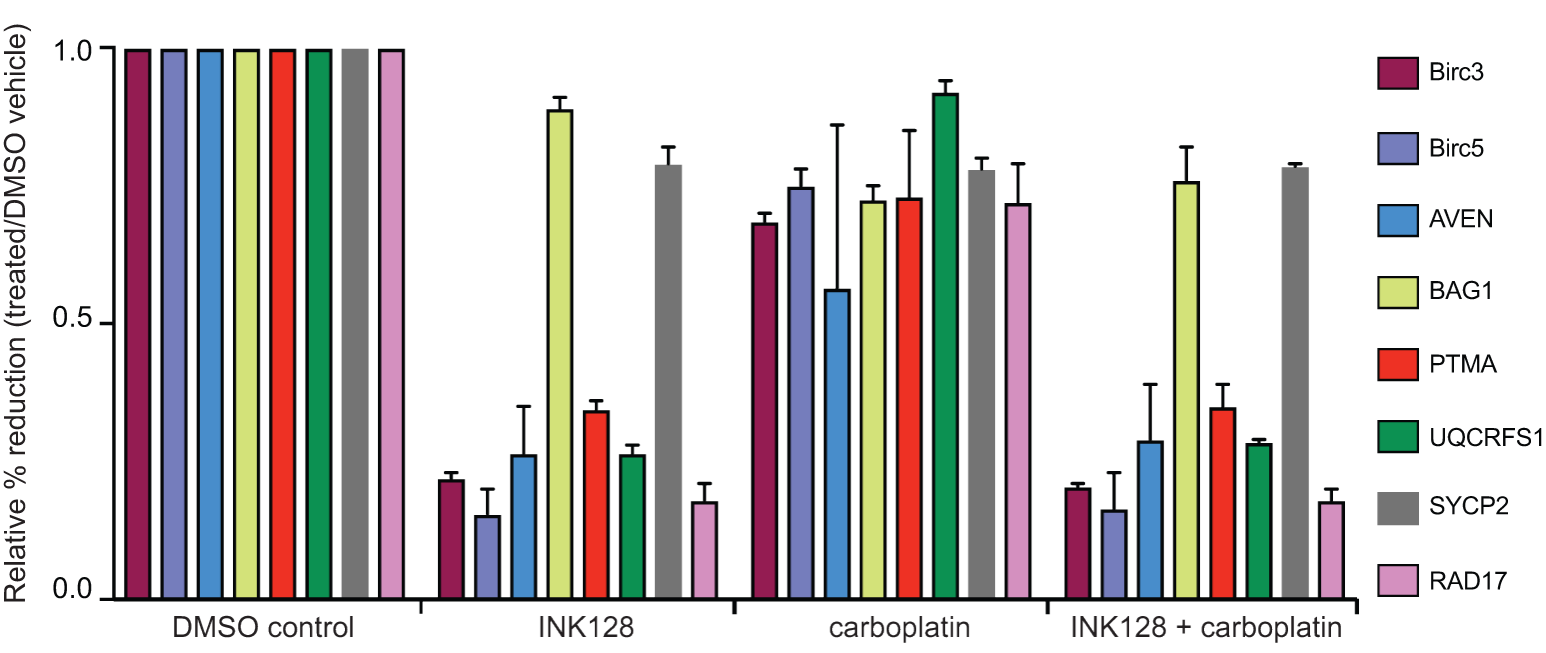
qRT-PCR analysis of select mRNAs in mTORC1/2 inhibited cells with and without carboplatin treatment Cells were treated at GI_50_ dose levels with 2 µM carboplatin for 5 h followed by 3 h or 18 h of 0.25 µM INK128 prior to cell harvest. Total mRNA and ≥4 ribosome heavy polysome mRNA was prepared as in Figure 3 legend. The abundance of selected mRNAs that encode proteins in the DNA damage response and survival response were assessed by qRT-PCR of the ≥4 ribosome (heavy polysome) fraction corresponding to well-translated mRNAs compared to total mRNA levels for each corresponding mRNA (see Methods for details). Results shown are the average of three independent experiments.

Independent confirmation of selective reduction in translated mRNAs was obtained by performing qRT-PCR analysis on total mRNA and heavy polysome fractions (≥4 ribosomes) for selected mRNAs identified in Tables 1 and 2 in a different carboplatin resistant ovarian cancer cell line, SKOV-3 cells. We first identified the carboplatin concentration that did not reduce SKOV-3 cell proliferation at 2 µM and the GI_50_ for INK128 at 0.25 µM. SKOV-3 cells were treated identically to OVCAR cells using 2 µM carboplatin and 0.25 µM INK128. Cells were harvested at 3 h and 18 h post-treatment with INK128 to determine the effect on selective mRNA translation downregulation and results averaged (Figure 5). We chose several mRNAs to test that were selectively downregulated in translation by mTORC1/2 inhibition (Birc3, Birc5, PTMA, UQCRFS1, AVEN, RAD17) compared to those that were relatively resistant or showed only a very small decrease (BAG1, SYCP2), comparing the ≥4 ribosome heavy polysome fractions to total mRNA. Significant reduction was again observed in the heavy polysome fraction compared to total mRNA levels for selected cell cycle and survival mRNAs compared to mRNAs in SKOV-3 cells, with the strongest reductions observed for BIRC3, BIRC5 and UQCRFS1 (∼75%) followed by RAD17 (∼60%). Control mRNAs relatively resistant to downregulation by mTORC1/2 inhibition in OVCAR-3 cells were also relatively resistant in SKOV-3 cells (BAG1, SYCP2).

## DISCUSSION

It has become clear that there is a role for inhibition of mTOR in ovarian cancer therapy [4, 32]. Several clinical trials have been initiated, but only with inhibitors of mTORC1 [14, 15]. There is therefore a need for greater investigation of dual mTORC1/2 inhibitors in ovarian cancer, particularly because they show efficacy in preclinical models. In some preclinical investigations, dual mTORC1/2 inhibitors have shown efficacy even as single agent therapy in endometrial cancer and platinum-sensitive ovarian cancer [23, 33]. Our investigation was focused on potentiating the anti-tumor effect of dual mTORC1/2 inhibitors with cytotoxic DNA damage chemotherapy and understanding the molecular mechanism by which the chemo-sensitizing synergistic effect of mTORC1/2 inhibition occurs.

Using genome-wide transcriptomic and translatomic analyses, we found a selective reduction in translation of specific mRNAs resulting from mTORC1/2 inhibition that sensitizes to genotoxic chemotherapy by impairing translation of mRNAs that promote increased cell survival and DNA damage and repair functions, and possibly mRNA translation functions (many ribosomal protein mRNAs were targets), all of which are involved in resistance to carboplatin-mediated cell killing. It was particularly interesting that the greatest number of mRNAs that were reduced in translation efficiency were found in the combined treatment set, despite the fact that carboplatin does not directly impair protein synthesis, which was shown to result solely from INK128 inhibition of mTORC1/2. Our data suggest that this is a result of moderately increased expression of mRNAs (< 2-fold) involved in the DNA damage and repair responses, as well as survival and cell cycle pathways, resulting from carboplatin treatment, whose translation is particularly sensitive to dual mTORC1/2 inhibition. Collectively, these mRNAs encode proteins that integrate DNA damage/repair responses, with cell cycle alignment, and anti-apoptotic effects by encoding proteins that prevent apoptotic cell death. For example, there were a number of mRNAs that were less than 2-fold increased by carboplatin treatment that function to promote the DDR and survival, but were strongly downregulation in translation by partial inhibition of mTORC1/2, such as caspase inhibitors and DNA polymerases. Our data supports a potential role for mTORC1/2 inhibitors in combination with carboplatin as therapy for patients with recurrent platinum-resistant high-grade ovarian cancer. Moreover, patients with tumors expressing high levels of pro-survival genes could benefit from the synergistic effect of this combination therapy.

## MATERIALS AND METHODS

### Cell culture

The platinum resistant ovarian cancer cell line OVCAR-3 [34] and SKOV-3 cell lines were obtained from the American Type Culture Collection (ATCC). Cells were authenticated by the ATCC using short tandem repeat (STR) profiling and used immediately. OVCAR-3 cells were derived from a patient with a platinum resistant recurrence of high-grade serous ovarian cancer with acquired resistance to platinum drugs [34] and at dose levels used in our study [35]. SKOV-3 cells were obtained from an epithelial-like high grade serous ovarian cancer resistant to platinum and adriamycin drugs [36]. Cells were cultured at 37°C, 5% CO_2_ in RPMI 1640 medium with L-glutamine and supplemented with bovine insulin at 0.01 mg/mL, penicillin/streptomycin at 10 mL/L, plasmocin at 50 µL/500 mL, and 20% fetal bovine serum (FBS). Cells were routinely tested and found to be mycoplasma free.

### Cell proliferation assay

The effect of INK128 on cell growth and proliferation, with and without carboplatin was assessed using the Promega CellTiter 96 Non-Radioactive Cell Proliferation Assay. Cells were plated 1000 cells/well in 96-well plates at 37°C, 5% CO_2_ for 24 h prior to drug treatment. The following treatments were carried out in triplicate: (1) DMSO control; (2) carboplatin at 1 µM; (3) INK128 at 0.25 µM; (4) carboplatin at 1 µM + INK128 at 0.25 µM. Colorimetric quantification was performed 24 h after seeding, and 24 h and 48 h after drug treatment.

### Clonogenic cell survival assay

600 cells were plated/well into 6 well plates and allowed to attach over 24 h, treated with DMSO as control, or 0.25 µM INK128 for 19 h followed by 1 µM or 2 µM carboplatin. Fresh media was replaced every 5 days without repletion of drug treatment, colonies grown for 12 days, washed with 1X PBS, fixed with 4% formalin and stained with 1% crystal violet. A colony was defined as consisting of at least 50 cells. Results are presented as the mean with standard error of the mean (SEM) based on 5 studies carried out in triplicate.

### Antibodies and immunoblot analysis

Following treatments, cells were washed twice in ice-cold PBS and lysed in RIPA buffer (150 mM NaCl, 50 mM Tris-HCl, pH 8.0, 1% NP-40, 0.5% sodium deoxycholate, 0.1% SDS, 1 mM EDTA, 1X Halt Phosphatase Inhibitor Cocktail [Thermo Scientific] and Complete Protease Inhibitor Cocktail [Roche]) at 4°C), lysates clarified by centrifugation at 13,000×*g* for 10 min and protein concentrations determined by DC Protein Assay (Biorad, Hercules, CA). Proteins were resolved by SDS-PAGE and transferred to PVDF membranes (Millipore). The phosphorylation status of most proteins was determined by immunoblotting the membrane first with phospho-specific antibody then stripping the membranes using Restore Western blot stripping buffer (Pierce), followed by re-probing the membranes with non-phospho-specific antibodies. Immunoblotting used the following antibodies, all from Cell Signaling Technology: rabbit anti-Akt (#9272), rabbit anti-phospho-Akt (S473) (#9271), rabbit anti-S6 (#2217), rabbit anti-phospho-S6 (S235/236), rabbit anti-4E-BP1 (#9452), rabbit anti-β-actin, all at 1:1000 dilution and Enhanced Chemiluminescence (ECL, GE Healthcare) used to detect protein signals as described by the manufacturer. All studies were carried out at least three times and representative immunoblots shown.

### Metabolic protein labeling and determination of protein synthesis rates

The effect of INK128 on protein synthesis was assessed by metabolic [^35^S]-Methionine incorporation, cells labeled with 25 µCi of [^35^S]-methionine/cysteine per mL (EasyTag Express Protein Labeling Mix, Perkin Elmer) in Met/Cys-free DMEM supplemented with gentamicin at 0.04 mg/mL, 5% FBS, and bovine insulin at 0.01 mg/mL, and incubated at 37°C for 30 min. Lysates were prepared using NP-40 buffer and specific activity of [^35^S]-methionine/cysteine incorporation into nascent protein was determined by trichloroacetic acid (TCA) precipitation onto GF/C filters and liquid scintillation counting. Studies were repeated three times and data presented as mean, normalized to the control, with SEM.

### Genome-wide transcription and translation studies

Cells (3x150 mm plates per condition) were treated with DMSO as control, 1 µM carboplatin, 0.25 µM INK128, 1 µM carboplatin + 0.25 µM INK128. Cells were treated with INK128 for 19 h prior to addition of carboplatin for 5 h. Plates with only carboplatin were treated at the same time point as the combination plates. Total combination drug treatment was 24 h. Growth media was removed, replaced with media containing 100 µg/ml cycloheximide, cells incubated for 10 min at 37°C, washed with ice cold 1X PBS supplemented with 100 µg/ml cycloheximide, cells harvested into ice cold 1X PBS supplemented with 100 µg/ml cycloheximide and complete protease inhibitor without EDTA (Roche), pelleted twice at 1000 rpm for 4 min, and resuspended in 750 µL polysome isolation buffer (200 mM Tris pH 7.5, 100 mM NaCl, 30 mM MgCl_2_). After 3 min, 250 µL detergent buffer (1.2% Triton N-101, 0.2 M sucrose in polysome isolation buffer) was added, cells were lysed with a Dounce homogenizer with 20 strokes, lysates clarified by centrifugation at 13,000x*g* for 10 min at 4°C and supernatants transferred to new tubes containing 100 µL heparin buffer (10 mg/mL heparin [Sigma-Aldrich], 1.5 M NaCl in polysome isolation buffer). RNA concentration of each sample was determined by NanoDrop and equal RNA amounts (400 µg/mL) layered onto 15-50% sterile sucrose gradients in polysome extraction buffer supplemented with 100 µg/mL cycloheximide. Gradients were centrifuged at 36,000 rpm for 2 h in a SW40 rotor (Beckman Coulter) at 4°C, equal fractions collected while simultaneously monitoring absorbance at 254 nm. 10 µL of 0.5 M EDTA were added to each fraction.

RNA was isolated from each fraction by extraction using the QIAGEN RNeasy MinElute Cleanup Kit. Fractions 7-14, representing high density polysomes, were combined and extracted. For normalization, an aliquot of total RNA was extracted from the same cell lysates used for polysome sedimentation and purified using the QIAGEN protocol. RNA quantity and quality for all samples were determined by bioanalysis (Agilent Technologies), and samples stored in nuclease-free water at −80°C. 10 µg of pooled RNA was used for microarray analysis using GeneChip Human Gene 2.0 ST Array (Affymetrix), according to the manufacturer instructions. Two complete sets of biologic replicates independently performed were used to develop transcriptome and translatome data sets for analysis.

Affymetrix chips were processed by the NYU School of Medicine Genome Technology Core and analyzed through the NYU School of Medicine Bioinformatics Core. Gene-level probeset summary of microarray data were obtained using the GCCN and SST transformation algorithms, RMA background correction, and quantile normalization provided in Expression Console Software, version 1.4.1 (Affymetrix). Control probesets and probesets lacking mRNA accession tags were removed from further analysis. To quantify translational efficiency, the difference in log_2_ intensity between matched polysomal RNA and total RNA was determined. To examine differences in transcription and translation, total RNA and polysome RNA were quantile normalized separately. Statistical analysis was performed using the limma R package [37].

### Assessment of mRNA levels by qRT-PCR

Forward and reverse primers were designed to detect the following mRNAs identified in Figure 5 with specific primer sequences available upon request. RNA was extracted from cells, cDNA was synthesized from the extracted and quantified sample of RNA (GoScript, Promega) and realtime PCR was performed in triplicate using SYBR green (Life Technologies) on a 7500 Fast-Dx RT-PCR Instrument (Applied Biosystems). The ∆CT was calculated using the 7500 software. Fold change values relative to the untreated corresponding control mRNAs were calculated and reported as graphs designed on GraphPad Prism.

### Statistical analyses

For clonogenic cell survival analysis, colony counts and colony diameter were multiplied to develop a colony burden score accounting for the total effect observed. Colony counts, diameters and their products were compared across treatment groups using non-parametric ANOVA methods. Paired tests used Student t-tests. Statistical analyses were performed using SPSS and GraphPad Prism software. Median survival was calculated for each treatment according to the Kaplan Meier method (SPSS version 21).

## SUPPLEMENTARY MATERIALS TABLES






