# Supplementary material for: mTORC1/2 inhibition re-sensitizes platinum-resistant ovarian cancer by disrupting selective translation of DNA damage and survival mRNAs
Source: Oncotarget. 2018 Sep 4;9(69):33064–76. doi: 10.18632/oncotarget.25869 (PMC6145695; doi:10.18632/oncotarget.25869)
Supplement: Supplementary file 1 [file oncotarget-09-33064-s001.pdf]

mTORC1/2 inhibition re-sensitizes platinum-resistant ovarian cancer by disrupting selective translation of DNA damage and survival mRNAs

SUPPLEMENTARY MATERIALS

For Supplementary Dataset 1 see attached excel file in Supplementary Files.

For Supplementary Dataset 2 see attached excel file in Supplementary Files.
